# Gliosis, misfolded protein aggregation, and neuronal loss in a guinea pig model of pulmonary tuberculosis

**DOI:** 10.3389/fnins.2023.1157652

**Published:** 2023-05-19

**Authors:** Amanda S. Latham, Charlize E. Geer, David F. Ackart, Isla K. Anderson, Kaley M. Vittoria, Brendan K. Podell, Randall J. Basaraba, Julie A. Moreno

**Affiliations:** ^1^Department of Environmental and Radiological Health Sciences, College of Veterinary Medicine and Biomedical Sciences, Colorado State University, Fort Collins, CO, United States; ^2^Brain Research Center, Colorado State University, Fort Collins, CO, United States; ^3^Department of Microbiology, Immunology, and Pathology, College of Veterinary Medicine and Biomedical Sciences, Colorado State University, Fort Collins, CO, United States; ^4^Department of Biomedical Science, College of Veterinary Medicine and Biomedical Sciences, Colorado State University, Fort Collins, CO, United States; ^5^Mycobacteria Research Laboratories, Department of Microbiology, Immunology, and Pathology, College of Veterinary Medicine and Biomedical Sciences, Colorado State University, Fort Collins, CO, United States; ^6^Center for Healthy Aging, Colorado State University, Fort Collins, CO, United States

**Keywords:** tuberculosis, neuroinflammation, glia, infection, neurodegeneration, tau, *Mycobacterium tuberculosis*, amyloid beta

## Abstract

Tuberculosis, caused by *Mycobacterium tuberculosis* infection, is an ongoing epidemic with an estimated ten million active cases of the disease worldwide. Pulmonary tuberculosis is associated with cognitive and memory deficits, and patients with this disease are at an increased risk for Parkinson’s disease and dementia. Although epidemiological data correlates neurological effects with peripheral disease, the pathology in the central nervous system is unknown. In an established guinea pig model of low-dose, aerosolized *Mycobacterium tuberculosis* infection, we see behavior changes and memory loss in infected animals. We correlate these findings with pathological changes within brain regions related to motor, cognition, and sensation across disease progression. This includes microglial and astrocytic proliferation and reactivity. These cellular changes are followed by the aggregation of neurotoxic amyloid β and phosphorylated tau and, ultimately, neuronal degeneration in the hippocampus. Through these data, we have obtained a greater understanding of the neuropathological effects of a peripheral disease that affects millions of persons worldwide.

## 1. Introduction

Tuberculosis (TB), resulting from aerosol infection by *Mycobacterium tuberculosis* (Mtb), is the thirteenth leading cause of death across the globe and the second leading cause of death due to an infectious agent (World Health Organization, 2022). TB is an ongoing global crisis; there are an estimated 10 million active cases of disease and 1.5 million deaths each year worldwide (Harding, 2020; Fukunaga et al., 2021). The COVID-19 pandemic has exacerbated the problem, with increased incidence of TB cases that have gone undiagnosed and untreated (Hogan et al., 2020; Jain et al., 2020; Migliori et al., 2020; Glaziou, 2021). Eradication of the disease is difficult; the bacteria are transmitted primarily through respiratory droplets from patients with active disease, and treatment requires a lengthy, intensive antibiotic regimen. Mtb primarily infects the pulmonary system, although it can also disseminate to other tissues, including the central nervous system (CNS) in rare cases. The immune response to Mtb is characterized by a massive influx of immune cells in an attempt to control infection, primarily macrophages and CD4^+^ T cells, followed by an overwhelming production of cytokines and chemokines, namely tumor necrosis factor (TNF) and interferon-gamma (IFNγ) (Ernst, 2012; O'Garra et al., 2013; de Martino et al., 2019).

Mtb infection has been widely researched, and the pulmonary immune response is well-documented, however, there is a gap in knowledge on how TB affects the CNS. Current research on the neurological impact of TB disease utilizes either *in-vitro* experimentation or *in-vivo* methods involving direct intracerebral injection of Mtb, excessive bacterial aerosol exposures, or models of tuberculosis meningitis (TBM) (Curto et al., 2004; Rock et al., 2005; Harris et al., 2007; Husain et al., 2008; Yang et al., 2009; Cannas et al., 2011; Green et al., 2011; Randall et al., 2014; Tripathi et al., 2014; Francisco et al., 2015; Mason et al., 2015; Qin et al., 2015; Tucker et al., 2016; Rohlwink et al., 2017; Othman et al., 2018; Lara-Espinosa et al., 2020; Xie et al., 2021). TBM is a progressive form of the disease where bacteria cross the blood–brain barrier (BBB) and cause inflammation of the meninges. Although these methods elucidate important findings related to mechanisms of neuronal infection and neuroinflammatory signaling in direct response to the bacteria, these studies do not translate to clinical settings. Diagnoses of TBM are rare, encompassing less than 2% of TB cases (Dodd et al., 2021). Physical brain infection or high bacterial inoculum models are ineffective representations of most disease cases, where infection occurs due to inhalation of a few aerosolized bacilli. Therefore, knowledge of the neurological changes that occur in response to solely peripheral infection is needed.

Epidemiologic studies show that patients with TB, without a diagnosis of TBM, are at an increased risk for Parkinson’s Disease (PD) and dementia (Frecker et al., 1994; Peng et al., 2015; Shen et al., 2016). Additionally, TB patients demonstrate decreased performance on evaluations of memory, cognition, and neuropsychological functioning (Robertson et al., 2018). It is also shown that patients who are coinfected with Human Immunodeficiency Virus (HIV) and pulmonary TB have decreased neuropsychological functioning and are three times more likely to experience cognitive impairment compared to uninfected patients, and nearly twice as likely as those who have HIV alone (Hestad et al., 2019). Such data demonstrate substantial risk for CNS-related symptoms associated with TB, despite Mtb rarely penetrating the brain. Accordingly, the permanent neurological implications of peripheral TB, independent of CNS infection, must not be overlooked.

Studies of other peripheral infections and diseases show evidence of a connection between CNS health and systemic inflammation. Patients with rheumatoid arthritis, a chronic inflammatory disease of the joints, have increased depression and anxiety, cognition decline, and markers of neuroinflammation compared to healthy individuals (Isik et al., 2007; Shin et al., 2012; Wallin et al., 2012). In another study, repeated systemic bacterial infection activated native microglia and increased synthesis of pro-inflammatory cytokines, including interleukin-1β (IL-1β), TNF, and interleukin-12 (IL-12). This occurred even after the infection was resolved (Püntener et al., 2012). Additional evidence in murine models of colitis demonstrates an altered expression of inflammatory modulators, including cyclo-oxygenase 2 (COX-2) and glial fibrillary acidic protein (GFAP), in the brain (Do and Woo, 2018). Moreover, peripheral inflammation is known to exacerbate symptoms of PD in both human patients and animal models, cause glial reactivity, and increases the synthesis of IL-1β in the prion-diseased brain (Combrinck et al., 2002; Machado et al., 2011; García-Domínguez et al., 2018; Süß et al., 2020; Chouhan et al., 2021). Similar findings are found in models of Alzheimer’s Disease (AD), where infection worsens cognitive decline and induces reactive glia (Cunningham et al., 2005; Perry et al., 2007; Lopez-Rodriguez et al., 2021). Research has also revealed a correlation between neurological state and peripheral inflammation; patients diagnosed with major depressive disorder have markedly increased peripheral blood levels of pro-inflammatory cytokines, including TNF, and interleukin-6 (IL-6) (Kim et al., 2007; Dowlati et al., 2010). These findings prove a relationship between systemic disease and health status of the CNS, further establishing the need for research on the neurological changes attributed to TB.

With 1.7 billion people infected worldwide, it is pertinent that we better understand the effects of TB on the brain to limit CNS damage and permanent neurological deficits in patients (Houben and Dodd, 2016). Although there are studies showing the long-term cognitive effects associated with Mtb infection, they are limited to cross-sectional designs and do not fully characterize neuropathology or evaluate the mechanism behind their findings. Here, we will assess TB-associated cognitive impairments and behavioral changes in an established low-dose aerosol guinea pig model of TB disease. We correlate these deficits to signs of neurotoxicity throughout the progression of disease; these include biomarkers of glial inflammation and neurodegeneration.

Glial reactivity is a universal characteristic of neuroinflammation and neurodegeneration, irrespective of the particular disease state (Skaper et al., 2018). Inflammation of the brain, or gliosis, is caused by phenotypically pro-inflammatory glial cells, including astrocytes and microglia. Although glia perform critical homeostatic functions in the brain, such as forming the BBB, maintaining synaptic neurotransmitters, regulating synaptogenesis, neuronal pruning, and immunological surveillance, they can also contribute to neuropathogenesis (Allen and Lyons, 2018; Giovannoni and Quintana, 2020). Activation and proliferation of glial cells by inflammatory signals and microbial components results in neurotoxic phenotypes. Neurotoxic microglia are more amoeboid, allowing for increased migration through dense parenchyma and produce pro-inflammatory molecules (Kreutzberg, 1996; Boche et al., 2013; Streit et al., 2014). Liddelow et al. established that microglia release interleukin-1α (Il-1α), TNF, and complement component 1q (C1q) upon activation, all of which play a crucial role in inducing neurotoxic astrocytes (Liddelow et al., 2017). Alternatively, activated astrocytes are more ramified, increasing contact with blood vessels and nearby cells, and upregulate expression of proteins such as GFAP (Eng and Ghirnikar, 1994; O’Callaghan and Sriram, 2005). Although the critical role microglia play in astrocytic polarization has been confirmed, additional research shows that low-level, early activation of astrocytes mediates microglial reactivity, which propagates the cycle of gliosis. Astrocytes promote microglial activation through the production of C-X-C motif chemokine ligand 10 (CXCL10), lipocalin-2 (Lcn2), and complement 3 (C3), as seen in models of stroke and epilepsy (Jha et al., 2019; Sanz and Garcia-Gimeno, 2020). This early astrocytic role may be understated, despite these cells comprising 60–70% of the total cells in the brain and that their contact with the microvasculature allows them to function as a first responder during altered brain states.

Glial inflammation and reactivity are determined not only by transcriptional expression and morphology, but also through quantity, proliferative state, and location within brain regions (Gómez-Nicola et al., 2013). Upregulation of S100 calcium-binding protein β (S100β) and GFAP indicate astrocyte activation. Together, this glial polarization results in the release of reactive oxygen species (ROS) and nitric oxide (NO), contributing to oxidative stress in neurons, and pro-inflammatory mediators (Chéret et al., 2008; Sheng et al., 2013). Chronic neuroinflammation disrupts synaptic function and causes irreversible damage to neurons, ultimately leading to the degeneration and death of these essential cells (Ransohoff, 2016).

Gliosis contributes to the misfolding of proteins, resulting in oligomers that form stable, insoluble aggregations. These aggregates are characteristic biomarkers of neurodegenerative diseases, including AD, PD, and dementia. One such protein is microtubule associated protein tau (Tau), which becomes phosphorylated in its misfolded form (pTau). Tau is normally involved in microtubule stabilization during axonal transport, and plays a role in DNA stabilization and synaptic functioning (Barbier et al., 2019). Phosphorylated residues have been identified and correlated to early and late stages of disease, and can aggregate into toxic neurofibrillary tangles. Similarly, amyloid beta is another physiologically relevant protein that is thought to play a role in neuroprotection from viral and bacterial pathogens. Due to improper cleavage events of the amyloid precursor protein (APP) by the gamma-lyase protease, the amyloid beta_1–42_ protein can accumulate into extraneuronal plaques (Lu et al., 2003). Another misfolded protein of interest in PD pathology is alpha-synuclein, which is found in phosphorylated and aggregated forms. The formation and accumulation of these proteins propagate increased misfolding (Frost et al., 2009; Brunello et al., 2020). This occurs, in part, due to the amplified production of enzymes and the native proteins themselves, to overcompensate for the loss of function. Misfolded proteins spread in a “prion-like” manner throughout various regions of the brain. This spread occurs through a cycle of intracellular formation and secretion followed by transcellular uptake by nearby cells (Wang et al., 2017; Brunello et al., 2020). Ultimately, the formation of these misfolded oligomers and aggregates results in reduced activity and survival of neurons, due to impaired physiological functioning by the proteins (Ciechanover and Kwon, 2015). Aggregates also exacerbate neuroinflammatory signaling events, which leads to endoplasmic reticulum stress and translational inhibition, which further contributes to neuropathogenesis (Ciechanover and Kwon, 2015; Currais et al., 2017).

In our study, animals are infected by low-dose aerosol of Mtb, similar to the natural route of infection in humans. Here, we demonstrate that this guinea pig model of Mtb exposure shows cognition and behavior changes that cannot be attributed to bacterial dissemination to the brain. Further, we characterize the induction and proliferation of pro-inflammatory glia throughout the progression of disease in multiple brain regions vital to physiological function, sensation, and cognition. Finally, we show neurodegenerative biomarkers and neuronal loss in animals. Although further investigation is necessary to fully elucidate the mechanism of neuropathogenesis, our data illuminate fundamental neurological changes in a rodent model that supports published epidemiological data.

## 2. Materials and methods

### 2.1. Animals and sample collection

Experiments consisted of 2- to 4-week-old, female, outbred Dunkin–Hartley guinea pigs (Elm Hill, USA). They were housed in a biosafety level 3 laboratory at the Colorado State University Laboratory Animal Resources facility accredited by the American Association for Accreditation of Laboratory Animal Care (AAALAC). All experimental animals were pair housed under constant temperature and humidity conditions (21° ± 2°C temperature and 30 ± 5% humidity). A 12-h light/12-h dark cycle was used, and animals had *ad libitum* access to food and water. Animals were monitored using a clinical scoring system for signs of morbidity and weighed weekly by laboratory staff for the duration of the experimental period. All animal experiments were performed in accordance with the National Research Council’s Guide for the Care and Use of Laboratory Animals and were approved by the Institutional Animal Care and Usage Committee (IACUC) at Colorado State University.

At the time of euthanasia, guinea pigs were administered 50 mg/kg of ketamine and 5 mg/kg of xylazine *via* intramuscular injection for anesthetic induction. Under terminal anesthesia, blood was collected, and then guinea pigs were euthanized by intraperitoneal overdose of pentobarbital. Tissues were collected for histopathology by fixing in 4% paraformaldehyde or 10% buffered formalin, or stored at −80°C for subsequent homogenization and quantification of Mtb.

### 2.2. Mtb aerosol exposure

Culture stocks of *Mycobacterium tuberculosis* (Mtb) strain H37Rv (TMC #102, Trudeau Institute) were collected at an OD600 nm between 0.8 and 1.0, and frozen at −80°C in Proskauer-Beck liquid medium containing 0.05% Tween-80. Titer was determined and bacteria were diluted in sterile water to 1 × 10^6^ colony-forming units (CFU)/mL. Animals were exposed to a low- dose of Mtb by aerosol, calibrated to deliver 20–50 bacilli per animal. Approximately 20 CFU of Mtb were delivered by aerosol to each animal with the Glas-Col Airborne whole-body exposure apparatus. Guinea pigs were exposed over the course of 2 days. Each run contained a single guinea pig for euthanasia and necropsy 24 h after exposure to confirm Mtb low-dose delivery to the lungs. Uninfected animals were exposed to sterile water using the same procedure in the Glas-Col device.

### 2.3. Behavioral testing

In order to minimize stress caused by the tests and discourage freeze behavior, all animals were handled daily 2 week prior to testing and throughout the duration of the experiments. Two total tests were performed: the Novel Object Recognition (NOR) Test and open field test. Tests were performed on randomized days, between 10 am and 7 pm, once every 2 weeks throughout the experimental period. Testing occurred under dim light in the same room as the animals were housed, separated by plastic drapes, to eliminate unnecessary stress caused by transfer to an alternate testing room. The researcher remained outside the testing room while trials occurred, and a white noise machine was used to alleviate the effects of background noise. Each test was recorded using a mounted GoPro HERO 5 and analyzed; behavior was scored blind to the treatment groups. All objects and chambers were thoroughly cleaned using Quatricide (diluted 1:64 in H_2_O; Pharmacal, Cat #: 65020F) followed by 70% EtOH between animals and decontaminated using Accel TB Disinfectant between groups.

### 2.4. Novel object recognition test

The Novel Object Recognition (NOR) Test is used to assess non-spatial, hippocampal, long- and short-term memory in animal models. Prior to testing, the guinea pigs were habituated to the chamber for 5 min per animal. The test consists of two periods: an acquisition phase and a testing phase. During the acquisition phase, the animals were placed in a chamber (24 inches × 24 inches × 18 inches) made of black acrylic with two of the exact same objects (A,A’) for ten min to familiarize themselves with them. Six hours later, the guinea pigs were placed in the same chamber with one of the same objects (A) from the acquisition period and one completely new object (B) for 5 min; animals were recorded for the entirety of the testing phase. The objects, approximately two inches wide by three inches tall, were constructed with Legos of various colors and shapes. They were placed approximately six inches apart in the apparatus. Data analysis was manually performed blind to the treatment groups by laboratory personnel. Time exploring the novel (b) and familiar (a) objects during the testing phase was determined, which only included direct contact the animal had with the object. This included, but was not limited to, biting, sniffing, or climbing on the object; any time the animal was not directly engaging with the object, such as standing near it, was excluded from the exploration time for that object. Total exploration time (e) was calculated by finding the sum of the exploration time for the familiar object (a) and novel object (b) (a + b). The absolute discrimination measure was calculated by subtracting the familiar object exploration time (a) from the novel object exploration time (b) (b – a). The discrimination index is evaluated as the novel object exploration time (b) minus the familiar object exploration time (a) divided by total exploration time (e) [(b – a)/e]. A positive discrimination index value means the animal spent more time exploring the unfamiliar object, which indicates that it remembers seeing the familiar object and has no memory loss or an intact long-term memory. Alternatively, a negative discrimination index means the animal spent more time exploring the familiar object and is indicative of non-spatial memory loss.

### 2.5. Open field test

Overall movement, locomotor activity, and anxiety-like behavior were determined using the open field test. The open field apparatus consisted of a square open field (24 inches × 24 inches × 18 inches) constructed of black acrylic. Twenty-four hours prior to testing, the guinea pigs were habituated to the chamber for 5 min per animal. Each guinea pig was taken from its home cage, placed into the center of the apparatus, and allowed to explore freely for a period of 5 min; animals were recorded for the entirety of the testing period. Video data were analyzed with Toxtrac (Rodriguez et al., 2018). Time spent in the interior portion of the apparatus is a standard way of identifying anxiety in animal models.

### 2.6. Bacterial burden/CFU counts

For confirmation of bacterial enumeration at 24 h and quantification of bacterial dissemination at each timepoint, lung, spleen, and brain were collected, weighed, and homogenized in PBS. Total liquid homogenate was diluted 1:10 and serial dilutions of tissue homogenate were performed in PBS and plated on nutrient 7H11 agar. Plates were incubated for 3–6 weeks at 37°C, CFU were counted, and CFU’s per gram of tissue were calculated.

### 2.7. Tissue processing for histopathology

Brains and visceral organs were extirpated *en bloc* and fixed whole in 10% buffered formalin or 4% paraformaldehyde at room temperature for at least 48 h. Tissues were processed using a Leica TP1020 Automatic Benchtop Tissue Processor and embedded in paraffin wax (Cancer Diagnostics, Cat #: EEPAR56). They were then sectioned on a Thermo Scientific HM 325–2 Manual Microtome at 5 μm thickness and mounted on positively charged glass slides (Superfrost Plus, Cancer Diagnostics, Cat #: 4951) for staining and analysis. One whole-tissue section per animal was stained with hematoxylin and eosin (H&E) for determination of morphological and histopathological changes.

### 2.8. Immunohistochemistry

Whole brain sections were stained for markers of gliosis and neurodegeneration. Tissue sections were deparaffinized in xylene and rehydrated through graded ethanol, followed by chemical and heat induced antigen retrieval using 0.01 M sodium citrate (pH 6.0) or EDTA buffer (1 mM EDTA disodium salt dihydrate, 0.05% Tween; pH 8.0) for 20 min at 100°C. This was followed by removal of endoperoxides by 0.3% hydrogen peroxide for 30 min at room temperature. Tissue was permeabilized [0.1% Triton-X in 1 M Tris Buffered Saline (TBS)] and blocking was performed in 10% goat, donkey, or horse serum diluted in 1 M TBS. Primary antibodies were diluted to their optimized concentrations in 1 M TBS and incubated on the tissue at 4°C overnight. A goat anti-ionized calcium binding adaptor molecule 1 (Iba1) antibody (1:400; Abcam, Cat #: ab5076) was used to identify microglia. A rabbit anti-S100 calcium-binding protein β (S100β) antibody (1:750; Abcam, Cat #: ab41548) was used for astrocyte identification. Amyloid beta was identified using an anti-beta Amyloid_1–42_ antibody (1:250; Invitrogen, Cat #: 44–344). Identification of phosphorylated tau was performed using the following antibodies: anti-phospho-Tau (Ser404) (1:400; Cell Signaling, Cat #: 35834), anti-phospho-Tau-T217 (1:200; ABclonal, Cat #: AP1233), and anti-phospho-Tau (Thr181) (1:800; Invitrogen, Cat #: MN1050). Neurons were identified using an anti-neuronal nuclei (NeuN) antibody (1:500; Cell Signaling, Cat #: 24307). Wash steps were performed using 2% bovine serum albumin and 2% Triton-X in 1 M TBS. An ABC HRP peroxidase detection kit (Vector Laboratories, Cat #: pk-4,000) and ImmPACT DAB Substrate, Peroxidase (HRP) Kit (Vector Laboratories, Cat #: sk-4,105) was used as chromogen and slides were counterstained with hematoxylin (Thermo Fisher Scientific, Cat #: 7231) and bluing solution (Cancer Diagnostics, Cat #: FX2107). All slides for each antigen of interest received the same immunoreaction period, which were visualized by a single pathologist in a blinded fashion. Slides were secured with a coverslip in mounting medium and stored at room temperature until imaging. Whole tissue images were taken using an Olympus BX53 microscope with an Olympus DP70 camera using an Olympus UPlanSApo 20x objective (N.A. = 0.75). Representative images were taken using an Olympus BX53 microscope with an Olympus DP70 camera using an Olympus UPlanFL N 40x objective (N.A. = 0.75).

### 2.9. Immunofluorescence

Paraffin embedded brain sections were deparaffinized in xylene and rehydrated through graded ethanol, followed by chemical and heat induced antigen retrieval using EDTA buffer (1 mM EDTA disodium salt dihydrate, 0.05% Tween; pH 8.0) for 20 min at 100°C. This was followed by permeabilization using 0.01% Triton X diluted in 1 M TBS. Blocking was performed with 2% donkey and goat serum diluted in 1 M TBS. Sections were stained for microglia using a goat anti-ionized calcium binding adaptor molecule 1 (Iba1) antibody (1:50; Abcam, Cat #: ab5076) and a donkey anti-goat Alexa Fluor 555 secondary antibody (1:500; Invitrogen, Cat #: A21432). Astrocyte stains used a mouse anti-S100 calcium-binding protein β (S100β) (1:750; Abcam, Cat #: ab212816) with a goat anti-mouse Alexa Fluor 555 secondary antibody (1:500; Invitrogen, Cat #: A21422) and a rabbit anti-glial fibrillary acidic protein (GFAP) (1:250; Dako, Cat #: Z0334) with a donkey anti-rabbit Alexa Fluor 647 secondary antibody (1:500; Invitrogen, Cat #: A31573). Following DAPI (Sigma), slides were mounted on glass coverslips in ProLong Gold Antifade mounting medium (ThermoScientific), fixed for 24 h at room temperature, and then stored at 4°C until imaging. Representative images were captured using an Olympus BX63 fluorescence microscope equipped with a motorized stage and Hamamatsu ORCA-flash 4.0 LT CCD camera using a 40x Olympus X-Apochromat air objective air objective (N.A. = 0.80). All slides quantified were imaged on the same day with the same exposure per channel.

### 2.10. Cellular quantifications

Whole-slide images of brain tissue stained for Iba-1 and S100β by immunohistochemistry were analyzed. Regions of interest were manually drawn for each brain region and number of positive cells was quantified using manual thresholding on the Count and Measure function of Olympus CellSens software (v1.18). Whole-slide images of brain tissue stained for GFAP by immunofluorescence was also analyzed. Regions of interest were manually drawn for each brain region, and mean gray intensity of GFAP expression for each ROI was determined using manual thresholding on the Count and Measure function of Olympus CellSens software (v1.18). Percent total expression or cell number was calculated for each protein of interest by determining the minimum (min) and maximum (max) quantifications for the data set. Each raw quantification (raw) for that brain region received the following calculation: [(raw – min)/(max – min)*100]. Slides stained for NeuN were imaged at 40× magnification (3–4 images per hippocampal region), and manually counted by a blinded scientist.

### 2.11. Pathological scoring

Whole slides stained for Amyloid β_1–42_ and phosphorylated tau were independently scored, in a blinded fashion, by three researchers. Positive immunohistochemical staining in the hippocampus and brain stem was designated a score between 0 and 5 based on the following factors: number of positive cells, amount of extracellular protein accumulation, and intensity of expression. A higher score equates to worse pathology. The mean of the three scores for each animal was calculated and represented.

### 2.12. Statistical analysis

All data were presented as mean +/− SEM. A ROUT (Q = 1%) outlier test was performed on all data to identify potential outliers, which were removed from the data set. Differences between experimental groups were analyzed using either an unpaired t-test with Welch’s *post hoc* correction or a one-way ANOVA with Tukey’s *post hoc* test. Statistical analysis was performed using Prism. Fit spline analysis was performed on glial data (200 segment output, 4–5 knots). Significance is denoted as * = *p* ≤ 0.05, ** = *p* ≤ 0.01, *** = *p* ≤ 0.001, and **** = *p* ≤ 0.0001.

## 3. Results

### 3.1. Low-dose aerosol with Mtb H37Rv failed to disseminate to the brain of guinea pigs

**Figure 1:**
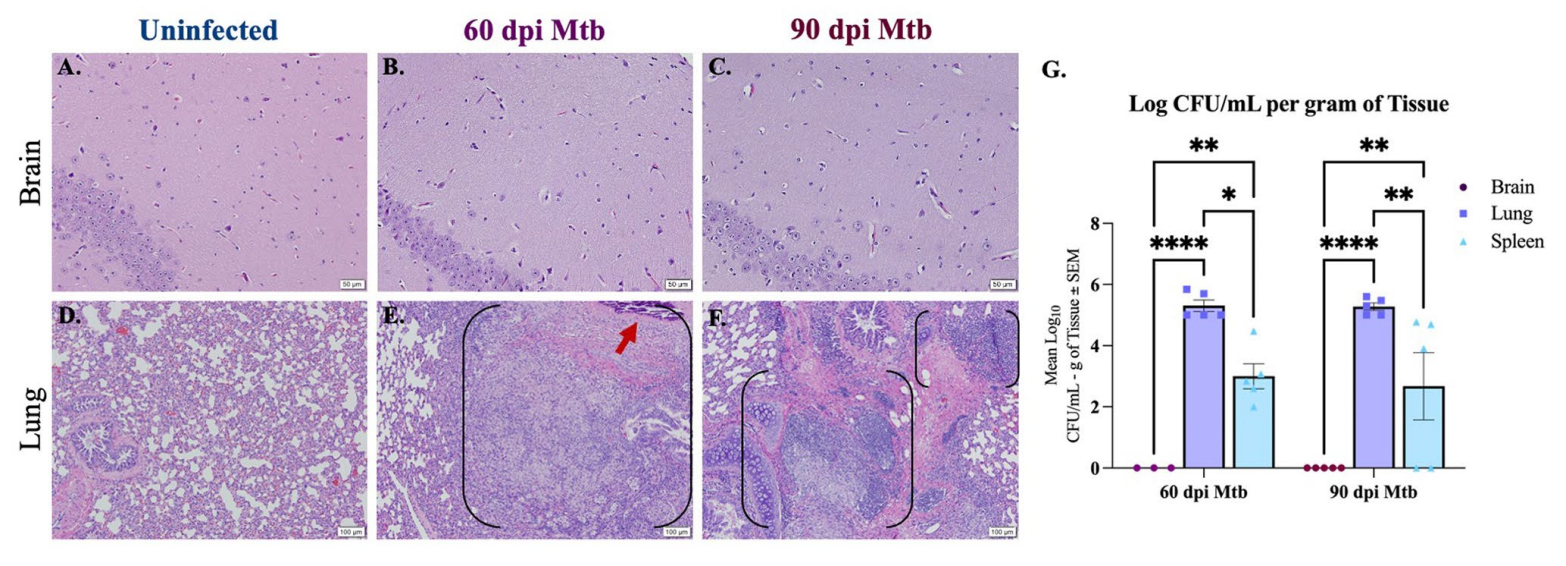
Lesions and bacterial colonies absent in brains of guinea pigs at 60 and 90 days post-infection. H&Es of brain show no lesions in animals uninfected **(A)** and infected with Mtb for 60 **(B)** and 90 days post-infection (dpi) **(C)**. Scale Bar = 50 μm. Lung histology shows granulomas (black brackets), including necrotic tissue and calcification (red arrow), in animals 60 and 90 dpi with a low-dose of Mtb by aerosol **(E,F)** but no granuloma formation is seen in uninfected animals **(D)**. Scale Bar = 100 μm. Colony-forming unit (CFU) assays indicate bacterial dissemination in lung and spleen homogenates, but no bacterial colonies were detected in brain at 60 or 90 dpi with Mtb **(G)**. The limit of detection for this assay is 2 CFU. Each bar represents the mean ± SEM (*N =* 3–5/group). One-way ANOVA analysis performed; *p ≤ 0.05, **p ≤ 0.01, ****p ≤ 0.0001.

Histopathology and colony-forming unit (CFU) assays were used to determine infection of peripheral organs and the brain in animals at 60 and 90 days post-infection (dpi). Lung, spleen, and brain tissue were collected from Dunkin-Hartley guinea pigs uninfected or infected with aerosolized Mtb H37Rv. Paraffin-embedded tissue sections were stained with hematoxylin and eosin (H&E) and examined. Representative hippocampal sections show no characteristic granulomatous inflammation, with an absence of cellular infiltration and microvascular abnormalities, in any of the animals infected for 60 and 90 days post-infection, similar to healthy tissue shown from uninfected animals (Figures 1A–C). Granulomatous lesions were not found in any brain region of all infected animals. H&E of lung from uninfected animals shows healthy vascular and pulmonary structure (Figure 1D). Lung tissue from Mtb-infected animals reveals granulomas characterized by areas of central necrosis, marked infiltration of peripheral immune cells, giant cells, and calcification (red arrow) across large areas of the pulmonary parenchyma (Figures 1E,F; between brackets). CFU assays show significant bacterial load in lung and spleen homogenates of animals infected for 60 and 90 days post-infection, but no colonies were detected from the brain homogenate of animals at either timepoint, which is indicative that detectable bacteria did not disseminate to the brain (Figure 1G). The limit of detection for this assay was 2 CFU.

### 3.2. Memory loss and hyperactivity evident in guinea pigs at 90 days post-infection

**Figure 2:**
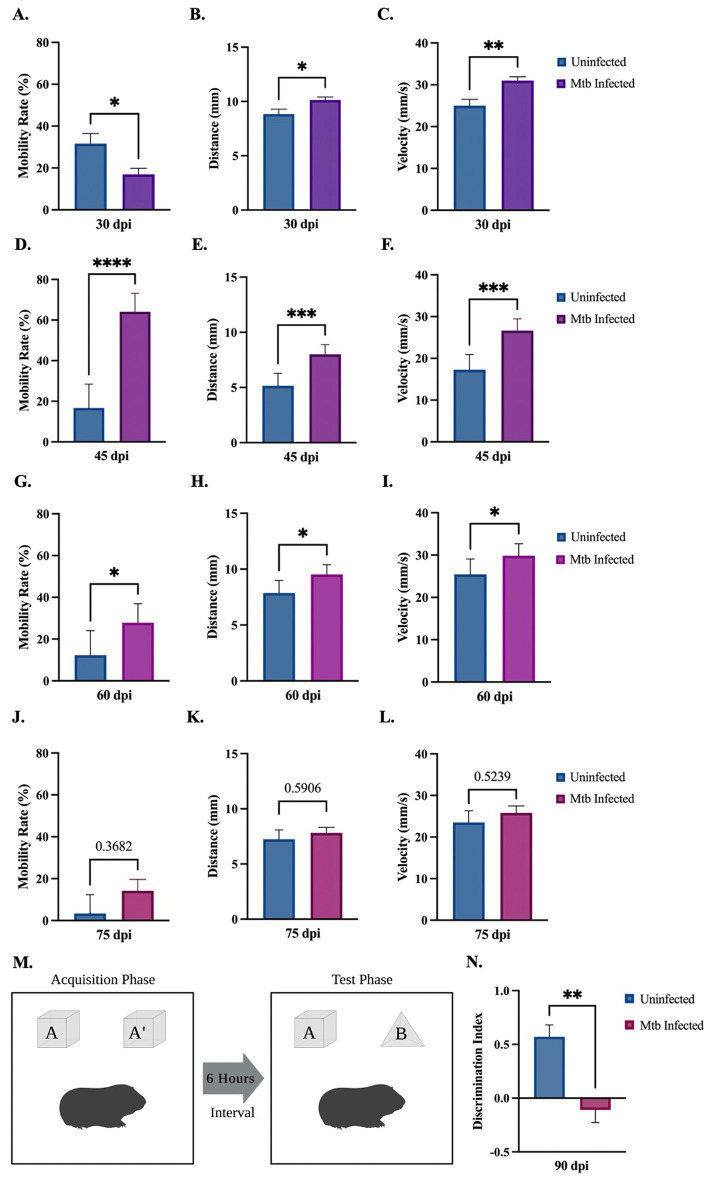
Behavioral deficits detected in guinea pigs at 90 days post-infection. Overall movement and cognition were evaluated in animals infected with Mtb compared to uninfected controls. The open field test assessed animal mobility rate, distance moved, velocity, and percent time spent in the interior of the apparatus over the progression of the disease. At 30 dpi a significant increase in distance and velocity was seen, although a decrease in mobility rate and time spent in the interior also occurred **(A–D)**. All readouts increase at 45 dpi **(E–H)** and 60 dpi **(I–L)** until 75 dpi, where no statistical difference is seen between groups **(M–P)**. Overall, these readouts show increased anxiety in infected animals at 45 and 60 dpi. Representation of the novel object recognition (NOR) test is provided **(Q)**. A significant decrease in discrimination index (DI) is demonstrated in animals by 90 dpi compared to controls, which is indicative of non-spatial memory loss. DI = (novel object exploration time – familiar object exploration time)/(total exploration time)  **(R)**. Each bar represents the mean ± SEM (*N =* 3–5/group). Unpaired *t*-test analysis performed; *p ≤ 0.05, **p ≤ 0.01, ***p ≤ 0.001, and ****p ≤ 0.0001. Created with BioRender.com.

Behavioral tests were performed to determine activity and loss of cognition in animals uninfected or infected with Mtb throughout the progression of the disease. The open field test was used to measure overall activity and mobility in animals. Although a decrease is seen at 30 dpi, Mtb-infected animals show a significant increase in mobility rate at 45 dpi and 60 dpi, and a trending increase at 75 dpi (Figures 2A,E,I,M). A significant increase in distance moved and velocity is also seen at 30 dpi, 45 dpi, and 60 dpi (Figures 2B,C,F,G,J,K). Animals spent more time in the interior portion of the apparatus at 45 and 60 dpi (Figures 2H,L). There was no difference in mobility rate, distance, velocity, or time spent in the interior in animals uninfected compared to those infected with Mtb for 75 dpi (Figures 2M–P). Together, these data suggest anxiety-like behavior associated with disease. The novel object recognition (NOR) test, which evaluates hippocampal memory, shows a significant decrease in discrimination index with progressive disease. This is indicative of non-spatial memory loss in infected animals (Figures 2Q,R).

### 3.3. Glial proliferation with progression of tuberculosis disease in multiple anatomical regions across the brain

**Figure 3:**
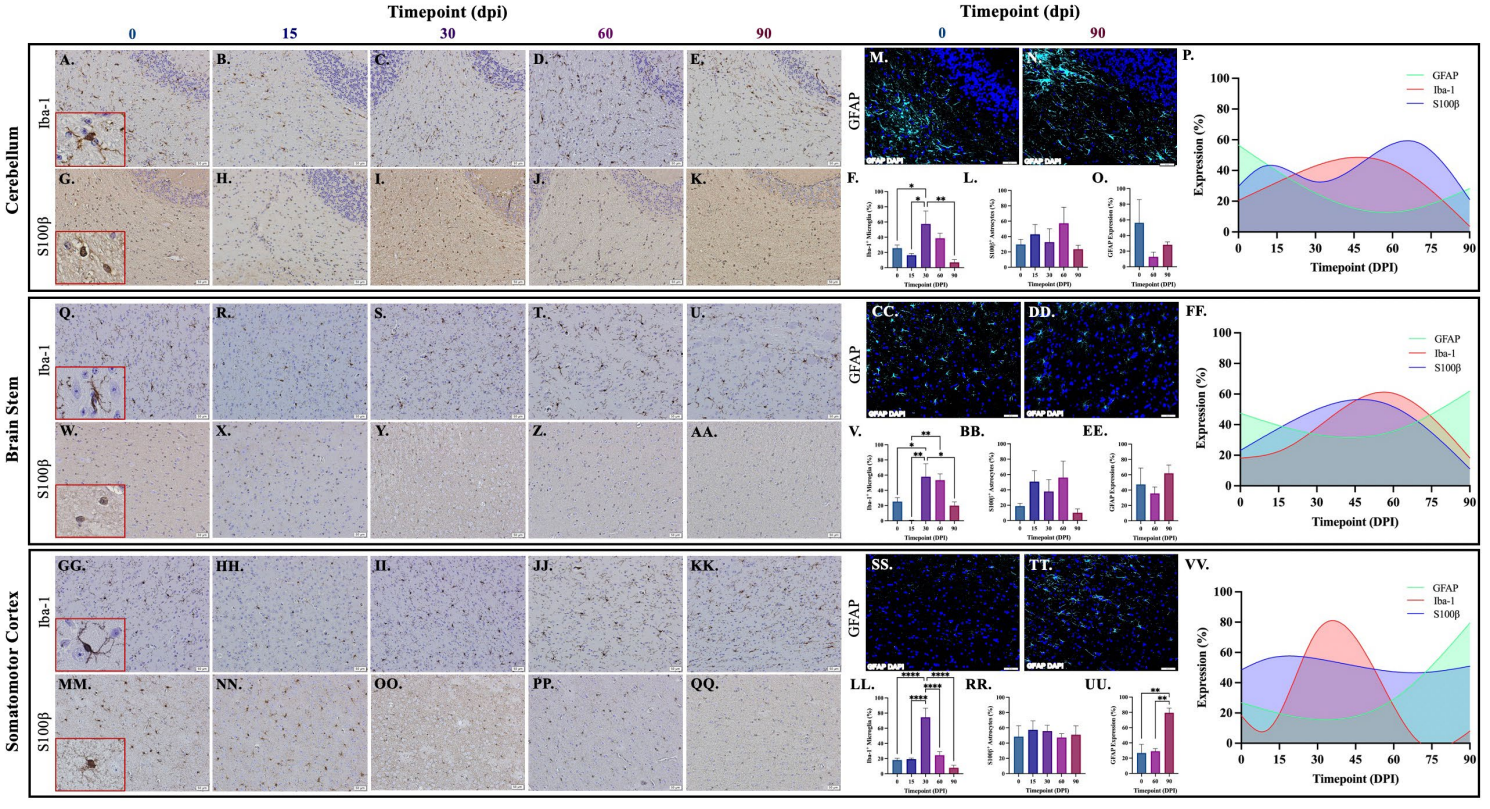
Mtb-associated gliosis in brain regions related to motor function. Immunohistochemical and immunofluorescent staining of guinea pig brain tissue for the microglial marker Iba-1 and astrocytic markers S100β and GFAP was performed. Glial cells in brain regions related to motor function were evaluated over the course of infection with aerosolized Mtb. Representative images of Iba-^1+^ microglia and S100β^+^ astrocytes at 0, 15, 30, 60, and 90 days post-infection (dpi) in the cerebellum **(A–E,G–K,M,N)**, brain stem **(Q–U,W–AA,CC,DD)**, and somatomotor cortex **(GG–KK,MM–QQ)** are shown. Iba-1^+^ cells significantly peak at 30 days post-infection then decrease by 60 and 90 dpi in the cerebellum **(F)**, brain stem **(V)**, and somatomotor cortex **(LL)**. S100β ^+^ cell numbers show a trending increase at 15 and 60 days post-infection in the cerebellum **(L)** and brain stem **(BB)**. No change in S100β ^+^ cells is seen in the somatomotor cortex **(RR)**, representative images of GFAP at time zero **(SS)** and 30dpi **(TT)**. No change in GFAP expression is seen in the cerebellum **(O)**, but a trending increase is found in the brain stem **(EE)** and significant increase in somatomotor cortex **(UU)** at 90 dpi. Graphs are shown to visualize the cellular changes of both microglia (Iba-1 in red) and astrocytes (S100β in blue and GFAP in green) in relation to one another across the progression of disease in the cerebellum **(P)**, brain stem **(FF)**, and somatomotor cortex **(VV)**. Each bar represents the mean ± SEM (*N =* 3–12/group). One-way ANOVA analysis performed; *p ≤ 0.05, **p ≤ 0.01, ***p ≤ 0.001, and ****p ≤ 0.0001. Scale Bar = 50 μm.

**Figure 4:**
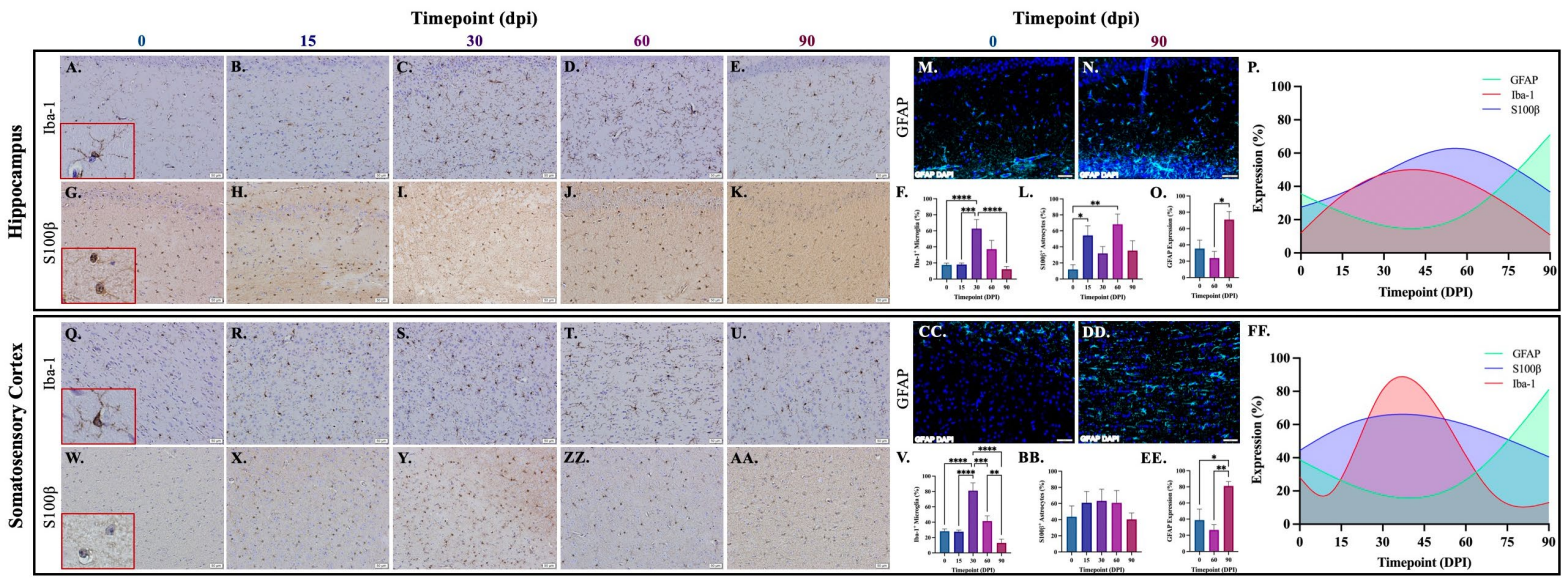
Mtb-associated gliosis in brain regions related to memory, cognition, and sensation. Immunohistochemical and immunofluorescent staining of guinea pig brain tissue for the microglial marker Iba-1 and astrocytic markers S100β and GFAP was performed. Glial cells in brain regions related to memory and cognition were evaluated over the course of infection with aerosolized Mtb. Representative images of Iba-1^+^ microglia and S100β^+^ astrocytes at 0, 15, 30, 60, and 90 days post-infection (dpi) in the hippocampus **(A–E,G–K,M,N)** and somatosensory cortex **(Q–U,W–AA,CC,DD)** are shown. Iba-1^+^ cells significantly peak at 30 days post-infection then decrease by 60 and 90 dpi in the hippocampus **(F)** and somatosensory cortex **(V)**. S100β^+^ cell numbers significantly increase at 15- and 60 days post-infection in the hippocampus **(L)** but no change is seen in the somatosensory cortex **(BB)**. GFAP expression significantly increases by 90 dpi in both brain regions **(O,EE)**. Graphs are shown to visualize the cellular changes of both microglia (Iba-1 in red) and astrocytes (S100β in blue and GFAP in green) in relation to one another across the progression of disease in the hippocampus **(P)** and somatosensory cortex **(FF)**. Each bar represents the mean ± SEM (*N =* 4–12/group). One-way ANOVA analysis performed; *p ≤ 0.05, **p ≤ 0.01, ***p ≤ 0.001, and ****p ≤ 0.0001. Scale Bar = 50 μm.

**Figure 5:**
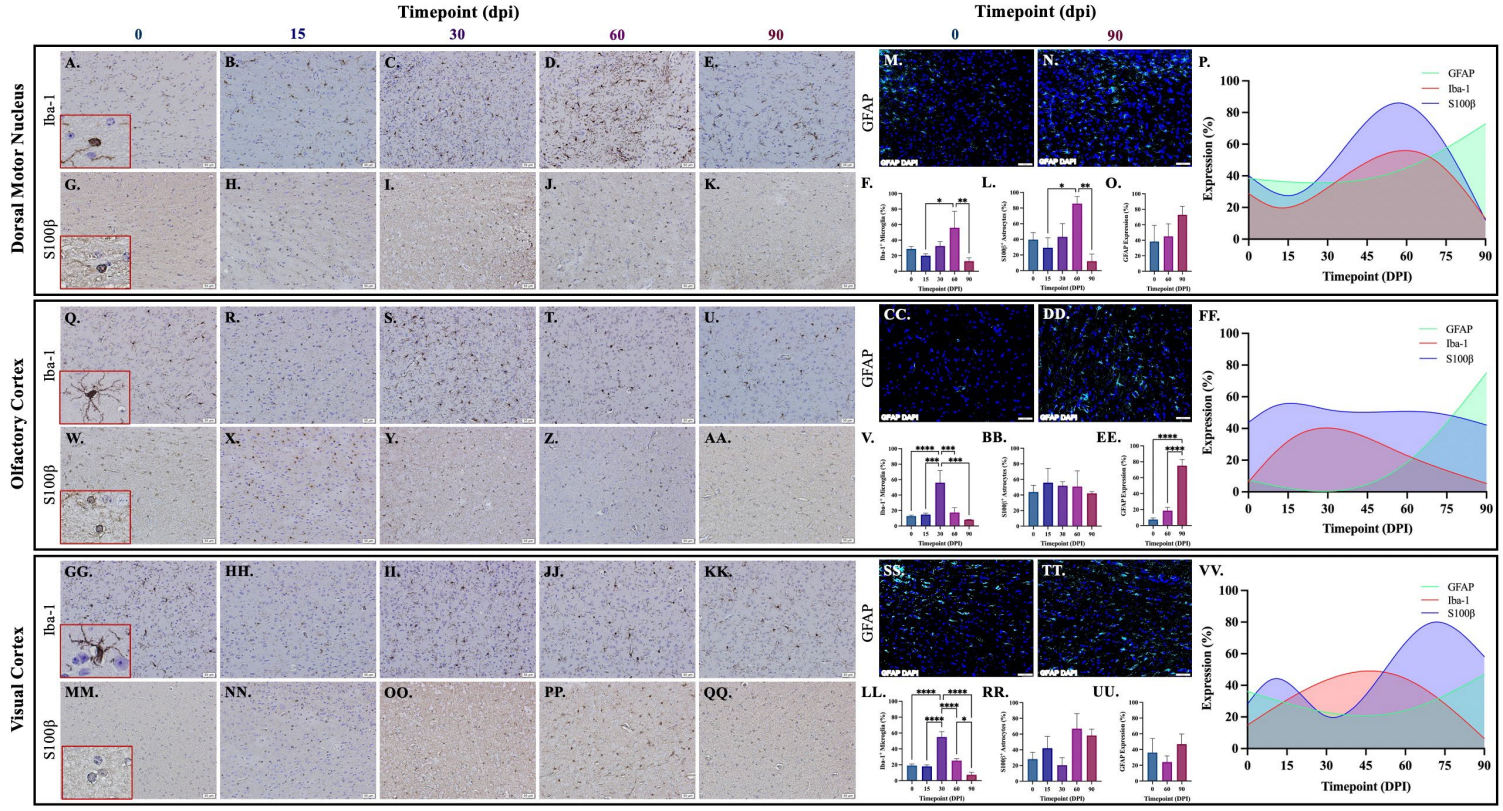
Mtb-associated gliosis in brain regions related to function. Immunohistochemical and immunofluorescent staining of guinea pig brain tissue for the microglial marker Iba-1 and astrocytic markers S100β and GFAP was performed. Glial cells in brain regions related to special senses and parasympathetic function were evaluated over the course of infection with aerosolized Mtb. Representative images of Iba-1^+^ microglia and S100β^+^ astrocytes at 0, 15, 30, 60, and 90 days post-infection (dpi) in the dorsal motor nucleus (DMN), where the vagus nerve innervates the brain **(A–E,G–K,M,N)**, olfactory cortex **(Q–U,W–AA,CC,DD)**, and visual cortex **(GG–KK,MM–QQ,SS,TT)** are shown. Iba-1^+^ cells significantly peak at 30 days post-infection then decrease by 60 and 90 dpi in the olfactory cortex **(V)** and visual cortex **(LL)**. Microglia in the dorsal motor nucleus peak later, at 60 dpi, before decreasing **(F)**. S100β^+^ cell numbers do not change in the olfactory cortex **(BB)** but a trending increase is seen at 60 dpi in the dorsal motor nucleus **(L)** and visual cortex **(RR)**. A trending increase in GFAP expression is found in the DMN **(O)** and significant increase at 90 dpi in the olfactory cortex **(EE)**. No change in GFAP expression is seen in the visual cortex **(UU)**. Graphs are shown to visualize the cellular changes of microglia (Iba-1 in red) and astrocytes (S100β in blue and GFAP in green) in relation to one another across the progression of disease in the DMN **(P)**, olfactory cortex **(FF)**, and visual cortex **(VV)**. Each bar represents the mean ± SEM (*N =* 3–12/group). One-way ANOVA analysis performed; *p ≤ 0.05, **p ≤ 0.01, ***p ≤ 0.001, and ****p ≤ 0.0001. Scale Bar = 50 μm.

Brains from guinea pigs infected for 0, 15, 30, 60, and 90 days post-infection with aerosolized Mtb were analyzed for glial migration and proliferation. Iba-1^+^ microglia as well as S100β^+^ and GFAP^+^ astrocytes were detected in brain regions critical for motor function, memory and cognition, and special senses. This included the following anatomical regions: cerebellum, brain stem, somatomotor cortex, hippocampus, somatosensory cortex, dorsal motor nucleus, visual cortex, and olfactory cortex. Microglial quantifications in the aforementioned regions, except for the dorsal motor nucleus, show a significant increase in Iba-1^+^ cells at 30 dpi that decreases back to baseline by 90 dpi (Figures 3F,P,V,FF,LL,VV, 4F,P,V,FF, 5V,FF,LL,VV). The dorsal motor nucleus, where the vagus nerve innervates the brain, shows an increase in microglia at 60 dpi that decreases by 90 dpi (Figures 5F,P). Representative images in the cerebellum (Figures 3A–E), brain stem (Figures 3Q–U), somatomotor cortex (Figures 3GG–KK), hippocampus (Figures 4A–E), somatosensory cortex (Figures 4Q–U), dorsal motor nucleus (Figures 5A–E), olfactory cortex (Figures 5Q–U), and visual cortex (Figures 5GG–KK) show these cellular fluctuations across progressive disease.

Astrocytes in those same brain regions were also quantified. S100β^+^ astrocytes peak at 60 dpi in the dorsal motor nucleus (Figures 5L,P). A similar finding is seen in other brain regions, except an increase in cell number at 15 dpi precedes the 60 dpi peak, in the cerebellum (Figures 3L,P), brain stem (Figures 3BB,FF), hippocampus (Figures 4L,P), and visual cortex (Figures 5RR,VV). Little to no change is seen in the somatomotor cortex (Figures 3RR,FF), somatosensory cortex (Figures 4BB,FF), or olfactory cortex (Figures 5BB,FF). Representative images in the cerebellum (Figures 3G–K), brain stem (Figures 3W–AA), somatomotor cortex (Figures 3MM–QQ), hippocampus (Figures 4G–K), somatosensory cortex (Figures 4W–AA), dorsal motor nucleus (Figures 5G–K), olfactory cortex (Figures 5W–AA), and visual cortex (Figures 5MM–QQ) show these cellular fluctuations across progressive disease. To see if astrogliosis is present in regions without changes in S100β^+^ cell number, or if glial reactivity progresses past 60 dpi, GFAP expression was quantified at 0, 60, and 90 days post-infection. Significant increases occur at 90 dpi in the somatomotor cortex (Figures 3UU,VV), hippocampus (Figures 4O,P), somatosensory cortex (Figures 4EE,FF), and olfactory cortex (Figures 5EE,FF) with trending increases in brain stem (Figures 3EE,FF) and DMN (Figures 5O,P). Graphical representations show the interaction between glia across the progression of the disease (Figures 3P,FF,VV, 4P,FF, 5P,FF,VV).

### 3.4. Glial reactivity is sustained as tuberculosis disease progresses

**Figure 6:**
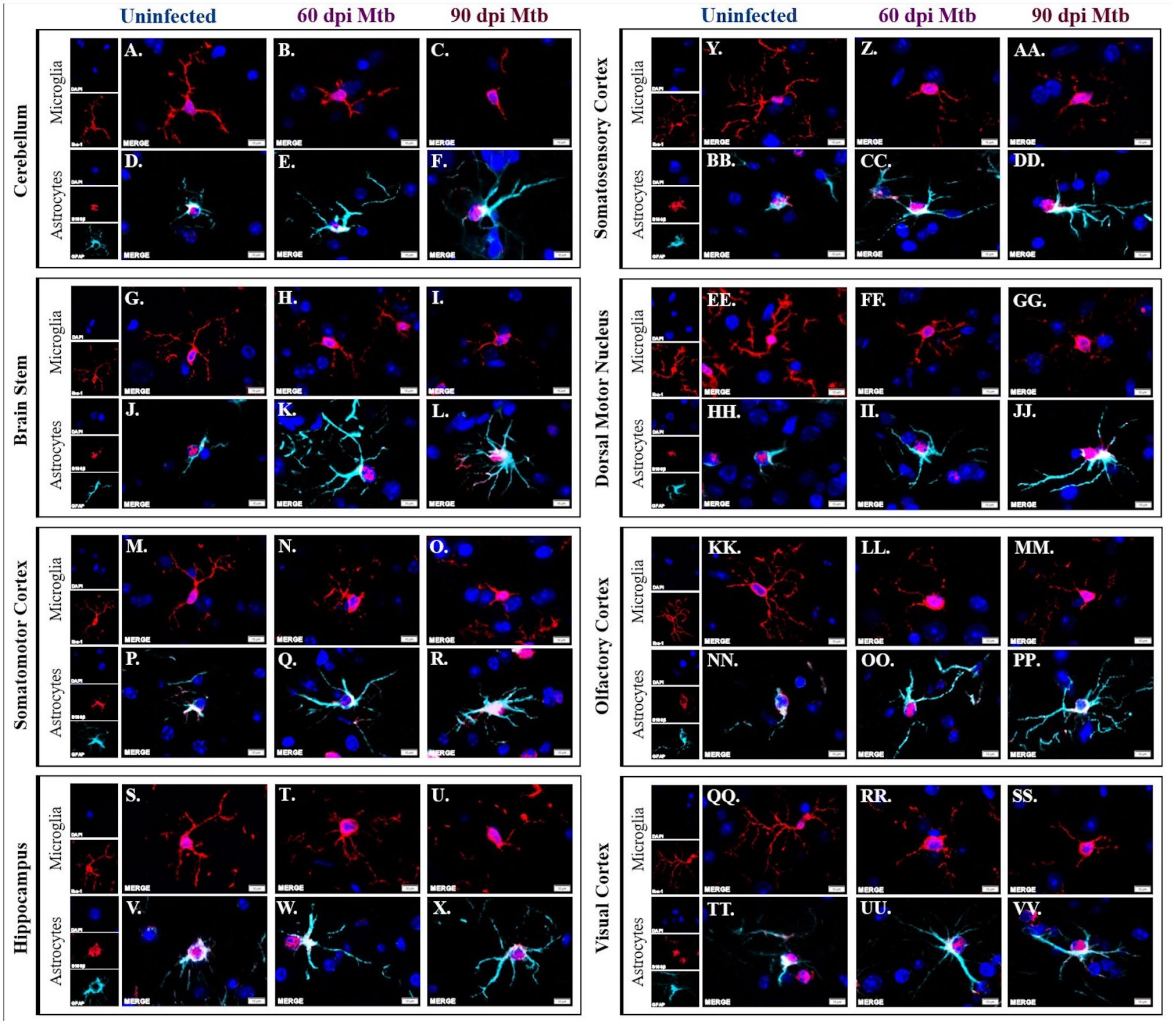
Activated glial morphology sustained throughout disease. Immunofluorescent staining of brain tissue was performed for identification of microglia, using an anti-Iba-1 antibody, and astrocytes, using anti-S100β and anti-GFAP antibodies. Representative images of cells in uninfected animals show phenotypically inactive microglia with increased branch density in the cerebellum **(A)**, brain stem **(G)**, somatomotor cortex **(M)**, hippocampus **(S)**, somatosensory cortex **(Y)**, dorsal motor nucleus **(EE)** olfactory cortex **(KK)**, and visual cortex **(QQ)**. Microglia in those same brain regions show ameboid-like, reactive microglial phenotypes 60 **(B,H,N,T,Z,FF,LL,RR)** and 90 days post-infection (dpi) **(C,I,O,U,A,AA,GG,MM,SS)**. Similarly, astrocytes in uninfected animals in those brain regions have an anti-inflammatory phenotype **(D,J,P,V,BB,HH,NN,TT)** whereas the cells 60 **(E,K,Q,W,CC,II,OO,UU)** and 90 days post-infection **(F,L,R,X,DD,JJ,PP,VV)** have reactive phenotypes. Scale Bar = 10 μm.

Brains from animals infected with Mtb for 0, 60, and 90 days were evaluated by immunofluorescence scanning microscopy for Iba1^+^ microglia and S100β^+^/GFAP^+^ astrocytes. Representative images of the eight brain regions stained above are shown, including the cerebellum, brain stem, somatomotor cortex, hippocampus, somatosensory cortex, dorsal motor nucleus, olfactory cortex, and visual cortex. Separated and merged channels from each brain region at 0 dpi show ramified microglia, or a non-reactive, anti-inflammatory cellular phenotype (Figures 6A,G,M,S,Y,EE,KK,QQ). Alternatively, pro-inflammatory microglia with an ameboid-like morphology are seen in all brain regions in animals 60 days post-infection (Figures 6B,H,N,T,Z,FF,LL,RR) and 90 days post-infection (Figures 6C,I,O,U,AA,GG,MM,SS). Similarly, separated and merged channels show non-reactive astrocytes, with fewer branches and shortened processes, at 0 dpi (Figures 6D,J,P,V,BB,HH,NN,TT) but activated astrocytes, characterized by increased branch density, are found in all brain regions 60 dpi (Figures 6E,K,Q,W,CC,II,OO,UU) and 90 dpi (Figures 6F,L,R,X,DD,JJ,PP,VV). Some astrocytes identified in the brains of Mtb-infected animals also come in contact with nearby cell nuclei (Figures 6E,F,K,L,Q,X,DD,II,JJ,OO,PP,UU,VV). The data establish that glial cells sustain neurotoxic phenotypes throughout the course of tuberculosis disease.

### 3.5. Amyloid beta aggregation presents in guinea pigs at 90 days post-infection with Mtb

**Figure 7:**
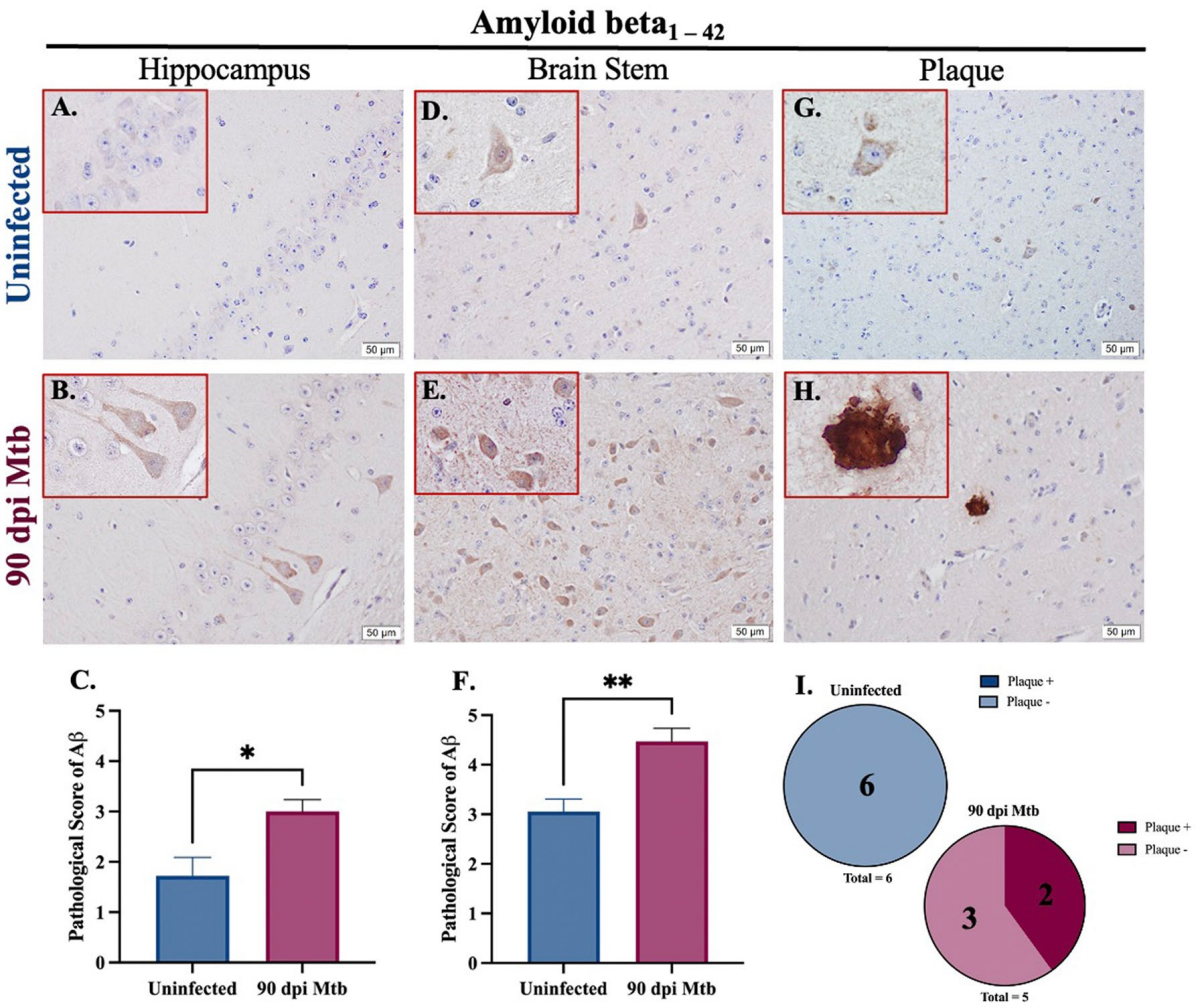
Amyloidosis in guinea pigs at 90 days post-infection with Mtb. Immunohistochemical staining of brain tissue was performed for identification of neurotoxic amyloid beta_1–42_. Positive staining was scored, as determined by number of amyloid β^+^ cells and degree of staining intensity, on a scale of 1–5. Increased intracellular amyloid β_1 – 42_ is seen in the hippocampus **(B)** and brain stem **(E)** of guinea pigs 90 dpi with Mtb compared to uninfected controls in those same brain regions **(A,D)**. Scoring of brain pathology shows a significant increase in misfolded protein accumulation in the hippocampus **(C)** and brain stem **(F)**. Extracellular plaques of amyloid beta are found in the brain stem of infected animals **(H,I)** but are absent in uninfected animals **(G,I)**. Each bar represents the mean ± SEM (*N =* 5–6/group). Unpaired *T*-test analysis performed; *p < 0.05, **p < 0.01. Scale Bar = 50 μm.

Amyloidosis was determined in guinea pigs infected with Mtb for 90 days. Representative images of intracellular amyloid β_1–42_ in the hippocampus (Figures 7A,B) and brain stem (Figures 7D,E) are shown. Additionally, representative images show extracellular aggregates identified in the brain stem of animals 90 dpi that are absent in uninfected animals (Figures 7G–I). Pathological scoring indicates amyloid β_1–42_ accumulation was significantly increased in both brain regions in animals 90 dpi compared to uninfected controls (Figures 7C,F).

### 3.6. Intracellular accumulation and extracellular tangles of hyperphosphorylated tau in guinea pigs at 90 days post-infection with Mtb

**Figure 8:**
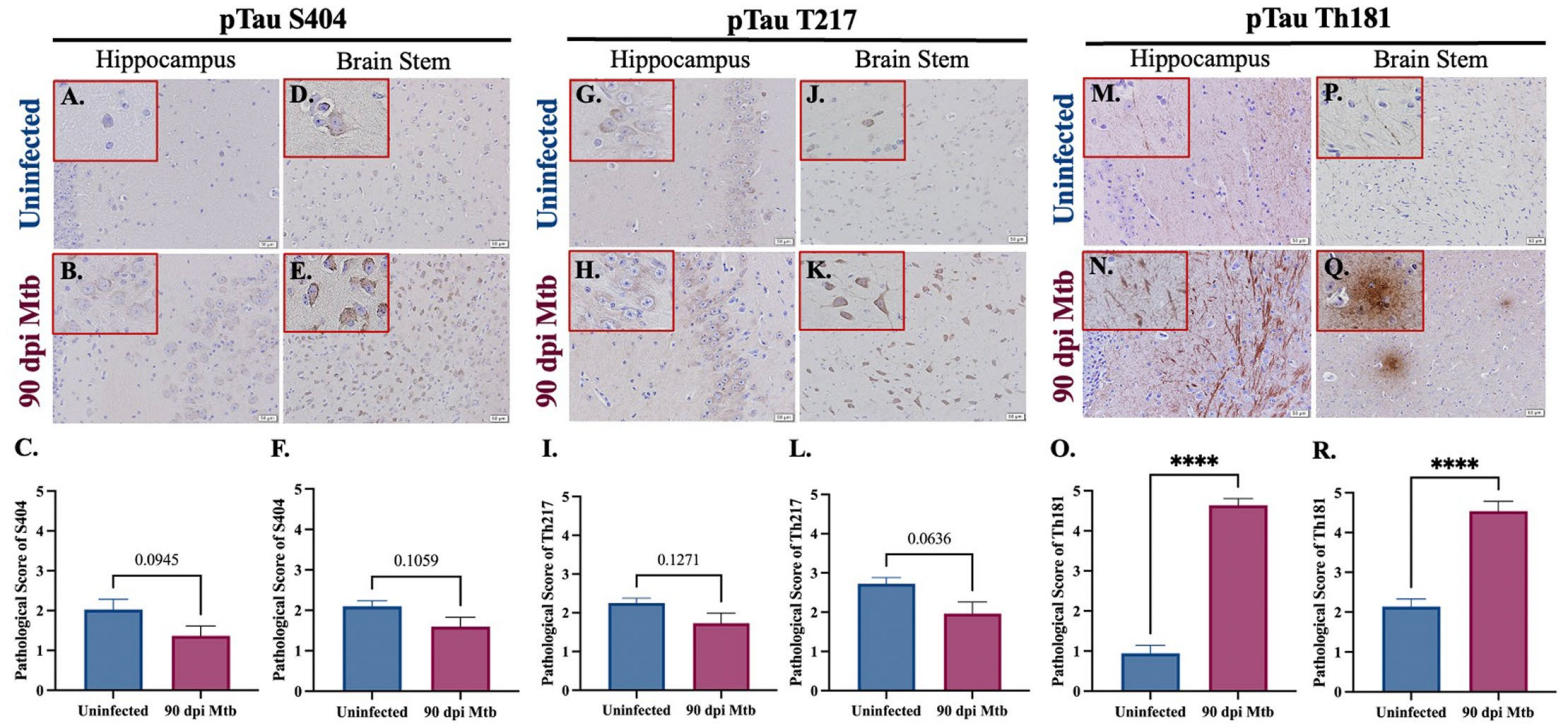
Hyperphosphorylated Tau and Tangles in Guinea Pigs at 90 days post-infection with Mtb. Immunohistochemical staining of brain tissue was performed for identification of tau phosphorylated at different amino acids. Positive staining was scored, as determined by number of tau^+^ cells and degree of intensity, on a scale of 1–5. No significant difference of tau phosphorylated at serine 404 was detected in the hippocampus **(B,C)** or brain stem **(E,F)** of guinea pigs at 90 dpi compared to uninfected controls in those same brain regions **(A,C,D,F)**. Similarly, tau phosphorylated at threonine 217 is identified in the hippocampus and brain stem **(G,H,J,K)**, although there is no significant change in pathological score in uninfected and infected animals **(I,L)**. An increase in fibrils of tau phosphorylated at threonine 181 is found in the hippocampus of guinea pigs 90 dpi **(N)** compared to uninfected controls **(M)**, as well as the formation of hyperphosphorylated tau tangles in the brain stem **(Q)** that are absent in uninfected animals **(P)**. Scoring of brain pathology shows a significant increase in misfolded protein accumulation in the hippocampus **(O)** and brain stem **(R)**. Each bar represents the mean ± SEM (*N =* 5–6/group). Unpaired *t*-test analysis performed; ns, not significant, ****p ≤ 0.0001. Scale Bar = 50 μm.

The presence of the neurodegenerative biomarker phosphorylated and aggregated tau was evaluated in Mtb-infected guinea pigs. Three different phosphorylation sites of tau (pTau) were analyzed by immunohistochemistry, including tau phosphorylated at serine 404 (pTau S404), threonine 217 (pTau T217), and threonine 181 (pTau Th181). Representative images and pathological scoring show no difference in pTau S404 and pTau T217 in both the hippocampus (Figures 8A–C,G–I) and brain stem (Figures 8D–F,J) of animals infected for 90 dpi compared to uninfected controls. There is a significant increase in the pathological score of pTau Th181 in both brain regions by 90 dpi (Figures 8O,R), including the formation of fibrils (Figure 8N) and hyperphosphorylated tau tangles (Figure 8Q) that are absent in uninfected animals (Figures 8M,P).

### 3.7. Neurodegeneration in multiple anatomical regions of the hippocampus in guinea pigs at 90 days post-infection with Mtb

**Figure 9:**
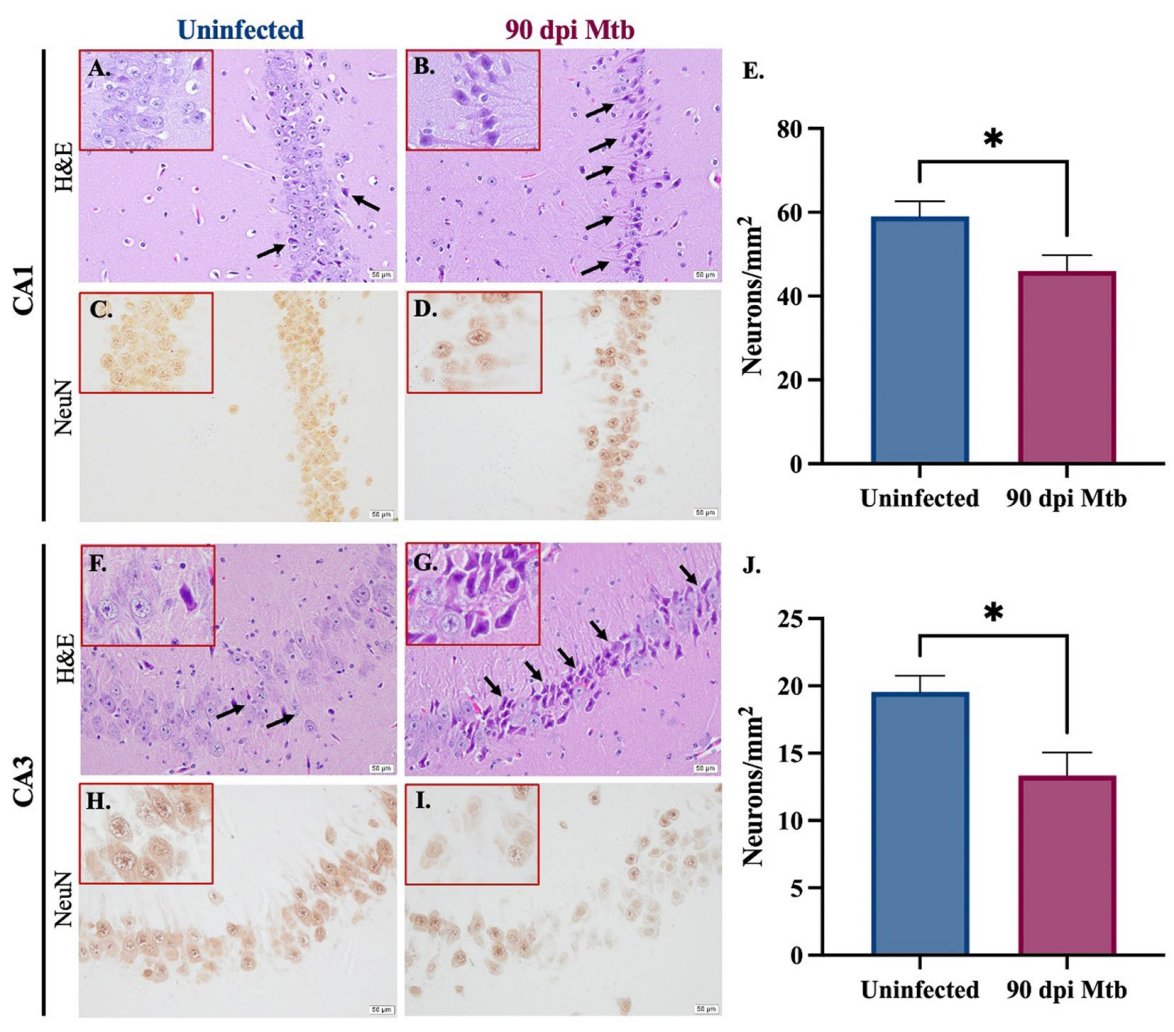
Neuronal loss and degradation in guinea pigs at 90 Days post-infection. H&E staining of the CA1 and CA3 anatomical regions of the hippocampus show an increase in pyknotic neurons (black arrows) in animals infected with Mtb for 90 dpi **(B,G)** compared to uninfected controls **(A,F)**. Quantification of immunohistochemical staining for neuronal marker NeuN shows a significant decrease in neurons in the CA1 **(C–E)** and CA3 **(H–J)** in animals infected for 90 days compared to uninfected animals. Each bar represents the mean ± SEM (*N =* 5–6/group). Unpaired *t*-test analysis performed; *p ≤ 0.05. Scale bar = 50 μm.

Staining was performed to determine neuronal loss and degradation in two relevant anatomical regions of the hippocampus, the Cornu Ammonis 1 (CA1) and Cornu Ammonis 3 (CA3). Paraffin-embedded tissue sections were stained with hematoxylin and eosin (H&E) and examined. Representative brain sections show an increase in the number of pyknotic neurons (identified as shrunken cells with condensed dark purple chromatin) in both hippocampal regions in guinea pigs infected with Mtb for 90 days (Figures 9A,B,F,G). Immunohistochemical staining for the neuronal marker NeuN was also performed, and representative images are shown (Figures 9C,D,H,I). Quantifications demonstrate a significant decrease in the number of NeuN^+^ neurons in the CA1 (Figure 9E) and CA3 (Figure 9J) regions of the hippocampus in Mtb-infected animals compared to uninfected controls.

## 4. Discussion

Pulmonary tuberculosis is associated with cognitive deficits and neurodegenerative disorders, establishing this infectious disease as an important risk factor for neurological impairment (Frecker et al., 1994; Peng et al., 2015; Shen et al., 2016; Robertson et al., 2018; Hestad et al., 2019). However, the pathology and cellular reactions in the brain associated with this disease are unknown. Our data are the first, to our knowledge, to fully characterize the cellular changes and etiology of neuropathology throughout the brain during this progressive inflammatory disease. Additionally, current experimentation relies on laboratory models and experimental methods that do not translate to clinical settings, such as the physical injection of bacteria into the brain or the use of Mtb-resistant murine models (Tucker et al., 2016; Lara-Espinosa et al., 2020). Research that appropriately translates to clinical disease is necessary in order to fully understand what is causing neurological detriments and, eventually, provide possible interventions to prevent them. Guinea pigs are not only susceptible to a variety of Mtb strains but exhibit pulmonary pathology and cellular immune responses similar to human disease (Ordway et al., 2007; Dharmadhikari and Nardell, 2008; Padilla-Carlin et al., 2008). In addition, their neurologically relevant proteins show a high degree of protein homology to that of humans (Supplementary Figure S2). Therefore, it is reasonable to infer that our guinea pig model of Mtb infection by low-dose aerosol closely mimics clinical disease in human patients.

Animals infected by aerosol with approximately 20 CFU of Mtb H37Rv bacteria established infection of peripheral organs, including the lung and spleen (Figure 1), without signs of morbidity or mortality or significant weight loss (Supplementary Figure S1). Despite evidence of bacterial dissemination, no characteristic granulomatous lesions were found in any brain region of the animals, and CFU assays did not detect bacteria in tissue homogenate (Figure 1). This indicates that the resulting neuropathogenesis is not a direct response to bacterial infection of the brain, but is instead a reaction to pulmonary and extrapulmonary disease, as is documented in patients with cognitive deficits that lack CNS infection (Frecker et al., 1994; Peng et al., 2015; Shen et al., 2016; Robertson et al., 2018; Hestad et al., 2019). While the CFU assay is the gold standard for quantifying bacterial load, it is recognized that unculturable or killed bacteria may not be detected by commonly used methods. Regardless, the absence of granulomatous lesions in the brains of all infected animals suggests that bacterial dissemination to the CNS did not occur, and it is within reason to consider these data as an effect of solely peripheral disease.

To determine whether cognition deficits similar to those documented in human patients are seen in our animal model, behavioral testing was performed on the Mtb-infected guinea pigs. This included the NOR test, which showed a decrease in hippocampal non-spatial memory, and the open field test, where animals exhibited anxious behavior, including increased mobility rate, distance moved, velocity, and time spent in the interior of the chamber (Figure 2). Combined, these data are consistent with clinical research and suggests damage to the brain that must be further investigated.

Neuroinflammation is an important pathological contributor to brain injury, disease, and homeostatic dysfunction (Skaper et al., 2018). Glial cells are critical mediators of this response; thus, the proliferation and reactivity of these cells were explored. In the cerebellum, brain stem, hippocampus, and visual cortex, a trending, although insignificant, increase in astrocyte number is seen as early as 15 dpi but decreases by 30 dpi in those same brain regions. Subsequently, a significant increase in microglia at 30 days post-infection in those same brain regions is observed. Similar to other disorders, this indicates that early activation of astrocytes occurs in the brain which, in turn, plays a role in initiating microglial reactivity (Figures 3–5; Jha et al., 2019; Sanz and Garcia-Gimeno, 2020).

Following the significant microglial migration and proliferation seen at 30 dpi, there is an exacerbated astrocytic response at 60 dpi in the cerebellum, brain stem, hippocampus, dorsal motor nucleus, and visual cortex. This highlights the interplay between these two glial cells, which has been established, where microglia play a role in astrocytic activation (Liddelow et al., 2017). Even though a change in S100β^+^ astrocytes is not found in all brain regions, including the somatomotor cortex, somatosensory cortex, and olfactory cortex, a significant increase in GFAP expression occurs in those regions at 90 dpi. Though changes in cellular quantifications indicate gliosis, astrocytes also upregulate GFAP when reactive and during neuroinflammation, supporting findings of astrogliosis in these brain regions (Hol and Pekny, 2015). This upregulation in GFAP expression is particularly relevant considering a change in astrocyte cell number is not seen in all models of neuroinflammation (Kamphuis et al., 2012). It must also be considered that glia have heterogenous roles and density depending on their brain region, making some areas more susceptible to glial reactivity than others. Cortical and hippocampal regions have high glial density and lower expression of immune activating genes, whereas the cerebellum and brain stem are less glia rich but proliferate at high rates (Tan et al., 2020). Altogether, these indications of gliosis throughout multiple brain regions, especially those related to motor function, explain the hyperactivity demonstrated by Mtb-infected animals during the open field test at the same post-infection timepoints (Mannix et al., 2014). While common in neurological research, Iba-1 is not solely a marker of microglia but is also present in macrophages. Therefore, these data set may also quantify infiltrating macrophages from the peripheral system. Optimization of antibodies against microglia-specific markers, such as transmembrane protein 119 (TMEM119), in guinea pigs could pose a potential way to address this in future studies. Furthermore, evidence reports that oligodendrocytes upregulate S100β protein in response to stress (Deloulme et al., 2004; Hachem et al., 2005; du et al., 2021; Smajić et al., 2021). It must also be considered that cellular quantifications may include oligodendrocytes, but additional GFAP expression data supplement our discovery of astrogliosis.

To further investigate the glial response to peripheral Mtb infection, morphological changes of the microglia and astrocytes in numerous brain regions were investigated. Neurotoxic glial phenotypes correlate to the production of pro-inflammatory molecules and reactive species that can compromise the brain over time. Astrocytes in guinea pigs 60 and 90 days post-infection appear to have an increased number and length of cellular protuberances compared to uninfected animals, which distinguishes a pro-inflammatory phenotype (O’Callaghan and Sriram, 2005). This change in cell arbor allows for amplified communication with nearby glia and neurons, as is seen in infected animals, where astrocyte processes come in contact with neighboring cell nuclei. Additionally, the microglia in these brain regions no longer have a ramified neuroprotective phenotype. Instead, they demonstrate decreased process length and branching, or an ameboid-like morphology, with cell body hypertrophy (Figure 6; Boche et al., 2013). Although a decrease in the cell number of both astrocytes and microglia are seen in the later stages of disease, the cells in those regions sustain glial reactivity as indicated by their morphology.

Glial priming, especially of microglia, causes these cells to become more sensitive to stimuli and plays a damaging role during age and in neurodegenerative disease (Perry and Holmes, 2014). This is seen in cases of neuroinflammation, which leads to the priming of microglia that contributes to AD later in life (Li et al., 2018). The prolonged reactivity of the glia found in the Mtb-infected brain could result in cellular priming that makes these cells more sensitive to additional stimuli and exacerbates their neurodegenerative effects, increasing susceptibility to environmental toxicants and age-related neurodegenerative disease later in life.

Aside from neuroinflammation, other mediators of neurodegenerative disease are misfolded proteins. Found in disorders such as AD, PD, and dementia, these proteins induce inflammatory signaling and lose their normal, but critical, function in neurons. Two neurotoxic proteins of interest are phosphorylated tau and amyloid beta_1-42_. Intracellular accumulation, as well as extracellular aggregates, of amyloid beta_1–42_ is identified in Mtb-infected guinea pigs (Figure 7). While the toxic effects of amyloid beta oligomers and plaques are disputed, it is believed that they are present in the AD brain, establishing their relevancy to the study of neurodegenerative effects. In addition to amyloid beta, tau phosphorylated at three amino acids is identified in animals at 90 dpi, which includes serine 404, threonine 217, and threonine 181. Both threonine phosphorylation sites are found in the proline-rich portion of the tau protein, whereas serine 404 is a component of the C-terminus (Noble et al., 2013). Phosphorylation at these sites decreases tubulin polymerization and microtubule affinity by the tau protein (Evans et al., 2000; Rajbanshi et al., 2023). Expression of only one residue, pTau Th181, is significantly increased in Mtb-infected guinea pigs, including the formation of extracellular aggregates; the other two phosphorylation sites have no significant change with exposure (Figure 8). Studies have shown that aggregation of misfolded proteins results in cellular senescence (Musi et al., 2018). Therefore, although there is no change in the phosphorylation of pTau S404 or pTau T217, the presence of extracellular tau tangles in infected animals reveals a worsened disease state. Research also postulates that even though pTau T217 may be a better diagnostic indicator, it is also found in intermediate to late stages of AD progression. In contrast, pTau Th181 is found in earlier, even pre-clinical, stages (Wesseling et al., 2020; Thijssen et al., 2021). Thus, increased accumulation of pTau T217 may occur in Mtb-infected guinea pigs as the disease progresses past 90 dpi. Additionally, misfolded alpha-synuclein, phosphorylated at serine 129, shows a trending increase in expression in the hippocampus and brain stem. Future experimentation of the substantia nigra, a critical region for PD neuropathology, is necessary to fully elucidate these effects (Supplementary Figure S3).

Finally, the effect of the reported neuroinflammation and misfolded protein accumulation on neurons was determined. Reactive species produced by pro-inflammatory glia reduce neuronal integrity and ultimately cause the death of these cells (Arimoto and Bing, 2003; Xie et al., 2004). Additionally, misfolded proteins, including amyloid beta and tau, result in loss of function and exacerbate inflammation, which further contributes to neurodegeneration. Animals infected with Mtb for 90 days show pyknotic neurons, cells characterized by nuclear condensation caused by necrosis or apoptosis. This is in combination with significant neuronal loss in two anatomical regions of the hippocampus, the Cornu Ammonis 1 (CA1) and Cornu Ammonis 3 (CA3) (Figure 9). In both regions, the CA1 especially, neurons are involved in forming, consolidating, and retrieving memories, which is decreased according to our behavior testing (Lee and Kesner, 2004). They are also implicated in neurodegenerative disease, especially in AD (WEST et al., 2000; Padurariu et al., 2012; Ugolini et al., 2018). Together, these detrimental neuronal effects are likely caused by the sustained glial reactivity and presence of aggregated neurotoxic proteins identified in that brain region (Figures 4, 7, 8). This cellular clearance and loss of neuronal function may explain the behavior changes and memory deficits demonstrated by Mtb-infected animals at 90 dpi (Figure 2). Although NeuN^+^ neurons decreased with infection, the function of NeuN is not entirely understood. This protein is found in the nucleus and perinuclear cytoplasm of neurons and plays an undetermined role in genetic regulation (Gusel'nikova and Korzhevskiy, 2015). Due to the uncertain function of NeuN, there may be neuronal disruptions that cannot be identified by immunohistochemical staining for the protein alone. However, loss of expression combined with morphological identification of pyknotic neurons definitively identifies degenerating cells.

In conclusion, pathological changes within the brain in a pertinent model of pulmonary tuberculosis assist in uncovering the cause of the cognitive deficits and neurodegeneration that is evident in human patients. Although these data play a key role in explaining the neuropathology associated with the disease, further research is necessary to reveal the mechanistic link between peripheral disease and neurological deficits. Here, we demonstrate that there is no detectible, replicating bacteria or granulomatous lesion formation within the brains of these animals, which illuminates an alternative route of cellular activation and ensuing neurodegeneration that cannot be attributed to Mtb itself. The glial response in the dorsal motor nucleus of the vagus nerve proposes a possible point of origin (Figure 5), especially considering the vagus nerve innervates organs affected by TB and is known to trigger neuroinflammation in response to peripheral stimulation (Steinberg et al., 2016; Yang et al., 2022). Additionally, the effects of the peripheral immune system may also play a pivotal, mechanistic role. Increased evidence indicates that the blood–brain barrier (BBB) is more permeable to peripheral immune cells, such as T cells, macrophages, dendritic cells, and B cells, than previously proposed. Due to the intense cellular reaction in the lungs occurring in response to Mtb infection, it is logical to hypothesize that infiltrating cells may play a role on the activation of glia in the brain, especially considering increased lymphocytes are seen in other models of systemic infection (Hickey et al., 1991; McManus et al., 2014). Although continued research is necessary, these data unravel important pieces of pulmonary TB-associated neuropathology.

## Data availability statement

The original contributions presented in the study are included in the article/Supplementary material, further inquiries can be directed to the corresponding author.

## Author contributions

JM and RB conceived and designed the project. BP, AL, and DA contributed to and/or performed *in-vivo* experimentation. AL, CG, DA, IA, and KV performed pathological experimentation and data analysis. AL, CG, and JM contributed to figure design. AL wrote the initial draft of the manuscript. AL, DA, RB, and JM edited the manuscript. All authors reviewed the manuscript prior to submission.

## Funding

This research received funding from the Colorado State University’s Center for Metabolism of Infectious Disease (C4MInD) and the Boettcher Webb-Waring Biomedical Research Award.

## Conflict of interest

The authors declare that the research was conducted in the absence of any commercial or financial relationships that could be construed as a potential conflict of interest.

## Publisher’s note

All claims expressed in this article are solely those of the authors and do not necessarily represent those of their affiliated organizations, or those of the publisher, the editors and the reviewers. Any product that may be evaluated in this article, or claim that may be made by its manufacturer, is not guaranteed or endorsed by the publisher.
