# Supplementary material for: Gliosis, misfolded protein aggregation, and neuronal loss in a guinea pig model of pulmonary tuberculosis
Source: Front Neurosci. 2023 May 19;17:1157652. doi: 10.3389/fnins.2023.1157652 (PMC10235533; doi:10.3389/fnins.2023.1157652)
Supplement: Supplementary file 1 [file Data_Sheet_1.PDF]

1 Supplementary Material

Method: Identification of Protein Homology

Analysis of protein homology between species was performed by aligning protein coding sequences for each neurological protein of interest. FASTA files for guinea pig (cavia porcellus) protein sequences were downloaded from the National Center for Biotechnology Institute's (NCBI) database and compared to the human (homo sapiens) and mouse (mus musculus) protein orthologs using the NCBI interface protein-protein Basic Local Alignment Search Tool (BLAST) for percent identity. This included S100 calcium-binding protein β (S100β) (cavia porcellus: S100 calcium-binding protein B; homo sapiens: protein s100-B; mus musculus: S100 protein, beta polypeptide, neural, isoform CRA), allograft inflammatory factor 1 (AIF1)/ionized calcium binding adaptor molecule 1 (Iba-1) (cavia porcellus: allograft inflammatory factor 1 isoform X1; homo sapiens and mus musculus: allograft inflammatory factor 1), glial fibrillary acidic protein (GFAP) (cavia porcellus and mus musculus: glial fibrillary acidic protein isoform X1; homo sapiens: glial fibrillary acidic protein isoform 1), microtubule associated protein tau isoform 1 (cavia porcellus and mus musculus: microtubule associated protein tau isoform X1; homo sapiens: microtubule associated protein tau isoform 1), and the amyloid beta precursor protein (APP) (cavia porcellus: amyloid beta precursor protein isoform X1; mus musculus: amyloid beta A4 precursor protein, isoform CRA; homo sapiens: amyloid precursor protein). An additional isoform of tau, isoform 4, was included because that is the antigen specific to the phosphorylated tau (pTau) antibodies used in this study (cavia porcellus and mus musculus: microtubule associated protein tau isoform X4; homo sapiens: microtubule associated protein tau isoform 4).

Method: Phosphorylated alpha-synuclein expression

Detailed process of immunofluorescent methods are included in the main manuscript. Brain sections were stained for phosphorylated α-synuclein using a mouse anti-phospho α-synuclein (Serine 129) (p129 α-synuclein) antibody (1:100; FUJIFILM Wako, Cat #: 015-25191) and a goat anti-mouse Alexa Fluor 647 secondary antibody (1:500; Invitrogen, Cat #: A32728). Neurons were stained using a rabbit anti-microtubule-associated protein 2 (Map2) antibody (1:100; Cell Signaling, Cat #: 4542) with a goat anti-rabbit Alexa Fluor 555 secondary antibody (1:500; Invitrogen, Cat #: A27039). Following DAPI (Sigma), slides were mounted on glass coverslips in ProLong Gold Antifade mounting medium (ThermoScientific), fixed for 24 hours at room temperature, and then stored at 4°C until imaging. Representative images were captured using an Olympus BX63 fluorescence microscope equipped with a motorized stage and Hamamatsu ORCA-flash 4.0 LT CCD camera using a 40x Olympus X-Apochromat air objective air objective (N.A. = 0.80). Whole-slide images of brain tissue stained for α-synuclein were analyzed; regions of interest were manually drawn for the hippocampus and brain stem and mean gray intensity of expression for each ROI was determined using manual thresholding on the Count and Measure function of Olympus CellSens software (v1.18). Percent total expression was calculated for each protein of interest by determining the minimum (min) and maximum (max) quantifications for the data set. Each raw quantification (raw) for that brain region received the

following calculation: ((raw – min)/(max – min))*100). All slides were imaged on the same day with the same exposure per channel.

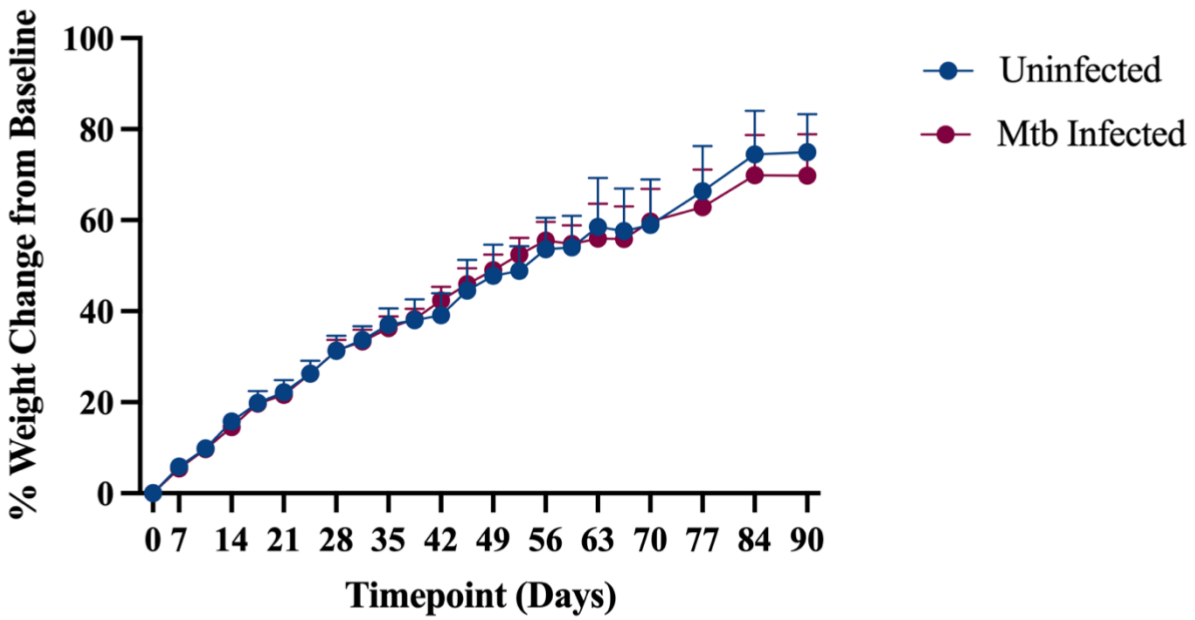

Supplemental Figure 1: No Signs of Morbidity or Mortality in Mtb-infected Animals.

Guinea pigs weighed once weekly for the duration of experimentation did not have any abnormal weight loss. Each point represents the mean ± SEM.

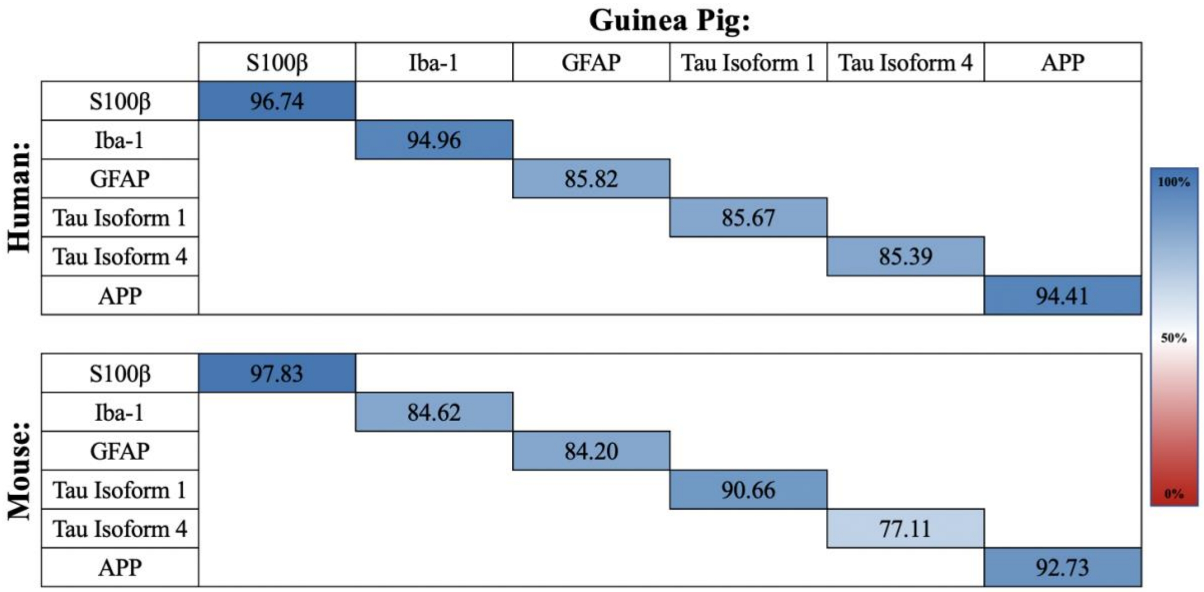

Supplemental Figure 2: Shared Homology of Neurological Proteins in Guinea Pigs, Humans, and Mice

Glial proteins S100β, Iba-1 (AIF1), and GFAP in guinea pigs are greater than 80% homologous with orthologs from humans and mice. Proteins involved in neurotoxic aggregation, tau and amyloid precursor protein (APP), in guinea pigs are also over 80% homologous with those in humans and mice, except for one isoform of tau which has a lower percent identity between guinea pigs and mice.

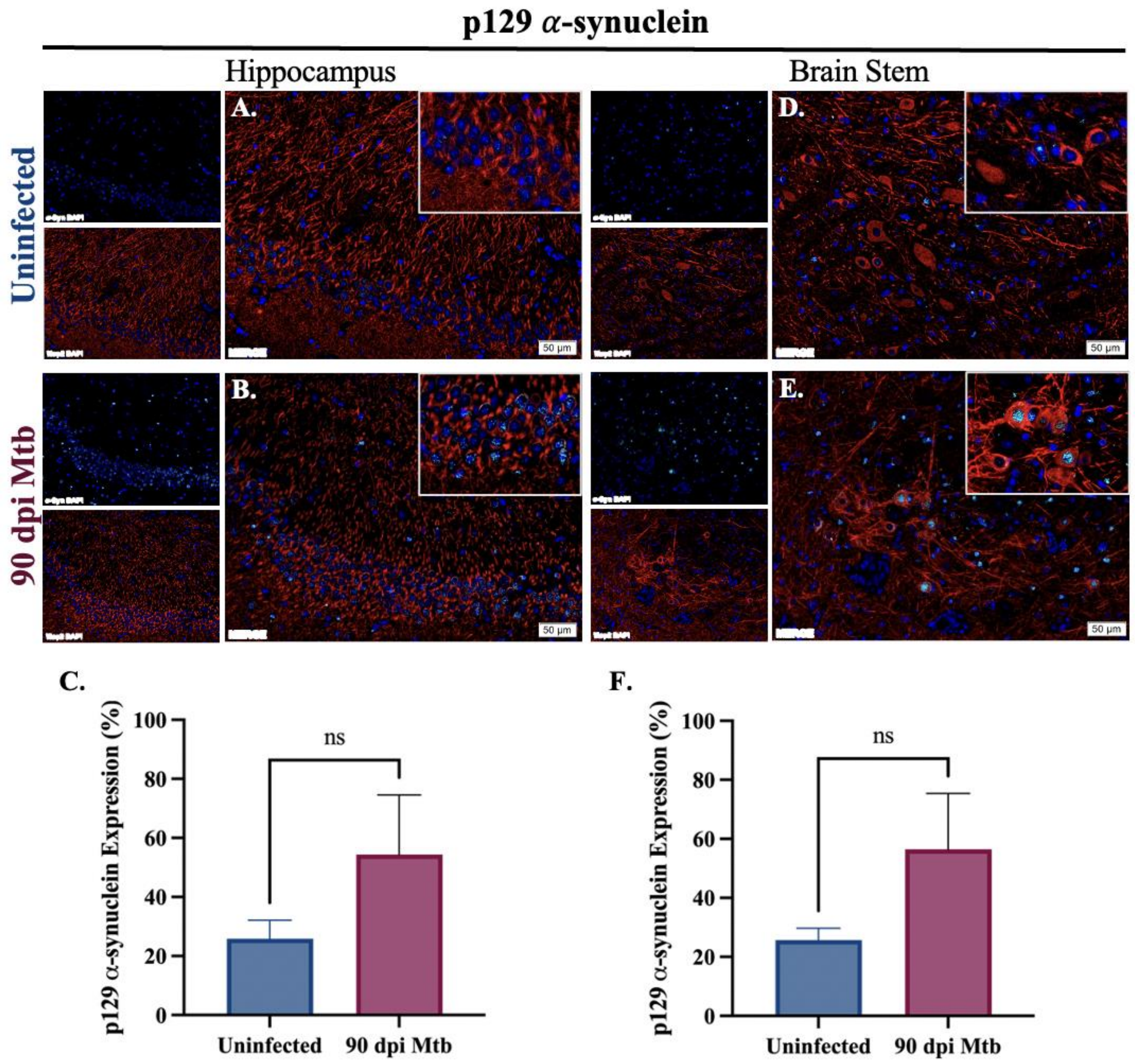

Supplemental Figure 3: Hyperphosphorylated α-synuclein Increased in Guinea Pigs at 90 days post-infection with Mtb.

Immunofluorescent staining of brain tissue was performed for identification of α-synuclein phosphorylated at serine 129. Fluorescent intensity was quantified for the hippocampus and brain stem. No significant difference was detected in both regions, although a trending increase in expression of phosphorylated α-synuclein is seen in the hippocampus (**B** and **C**) and brain stem (**E** and **F**) of guinea pigs at 90 dpi compared to uninfected controls in those same brain regions (**A** and **C; D** and **F**). Each bar represents the mean ± SEM ($N = 5 – 6$/group). Unpaired t-test analysis performed; ns = not significant. Scale Bar = 50 μm.
